# A Physical TCAD Mobility Model of Amorphous In-Ga-Zn-O (a-IGZO) Devices with Spatially Varying Mobility Edges, Band-Tails, and Enhanced Low-Temperature Convergence

**DOI:** 10.3390/mi15070829

**Published:** 2024-06-27

**Authors:** Mischa Thesberg, Franz Schanovsky, Ying Zhao, Markus Karner, Jose Maria Gonzalez-Medina, Zlatan Stanojević, Adrian Chasin, Gerhard Rzepa

**Affiliations:** 1 Global TCAD Solutions (GTS) GmbH, 1010 Vienna, Austria; 2 Imec, 3001 Leuven, Belgium

**Keywords:** indium gallium zinc oxide, TCAD, a-IGZO, semiconductor modeling, amorphous semiconductors, disordered materials

## Abstract

Amorphous indium gallium zinc oxide (a-IGZO) is becoming an increasingly important technological material. Transport in this material is conceptualized as the heavy disorder of the material causing a conduction or mobility band-edge that randomly varies and undulates in space across the entire system. Thus, transport is envisioned as being dominated by percolation physics as carriers traverse this varying band-edge landscape of “hills” and “valleys”. It is then something of a missed opportunity to model such a system using only a compact approach—despite this being the primary focus of the existing literature—as such a system can easily be faithfully reproduced as a true microscopic TCAD model with a real physically varying potential. Thus, in this work, we develop such a “microscopic” TCAD model of a-IGZO and detail a number of key aspects of its implementation. We then demonstrate that it can accurately reproduce experimental results and consider the issue of the addition of non-conducting band-tail states in a numerically efficient manner. Finally, two short studies of 3D effects are undertaken to illustrate the utility of the model: specifically, the cases of variation effects as a function of device size and as a function of surface roughness scattering.

## 1. Introduction

Amorphous indium gallium zinc oxide (a-IGZO) is emerging as an important new amorphous semiconductor material, both in conventional applications, such as thin-film transistors (TFTs) for visual displays [1], and in emerging applications, such as 3D DRAM [2]. All of these applications require a clear understanding of transport physics in a-IGZO and physically accurate modeling of carrier mobilities, which differ notably in their behavior from both conventional semiconductors such as silicon and from other amorphous semiconductors like amorphous silicon (a-Si).

However, although the field of mobility modeling of a-IGZO is very active, efforts almost exclusively focus on “compact” models rather than TCAD. Note that by “compact” models, we mean an effectively zero-dimensional description that takes in a voltage and returns a mobility using, for example, a numerical integration in energy rather than a compact analytical expression specifically intended for circuit design. Although initially there was a great deal of debate [3,4,5,6] as to the basic transport mechanisms dominant in a-IGZO, the bulk of current “compact” modeling research [3,7,8,9,10,11,12,13] has largely settled on a certain agreed-upon conceptual picture. This picture envisions a-IGZO as having a conduction band-edge or so-called “mobility edge”, $E_{b}\left(r\right)$, that spatially varies throughout the system such that its randomized value obeys a certain probability distribution. The probability distribution, $P\left(E_{b}\right)dE_{b}$, represents the fraction of the total system volume with a band-edge between $E_{b}$ and $E_{b}+dE_{b}$ and is usually assumed to have a Gaussian form:

(1) $$P\left(E_{b}\right)=\frac{1}{\sqrt{2\pi \sigma_{E_{b}}^{2}}}\exp\left(\frac{{\left(E_{b}-\langle E_{b}\rangle \right)}^{2}}{2\sigma_{E_{b}}^{2}}\right)$$

where $\langle E_{b}\rangle$ and $\sigma_{E_{b}}$ represent the mean and standard deviation, respectively, of the band-edge. The justification of this Gaussian assumption stems from some of the earliest work on disordered semiconductors by, most notably, E. O. Kane [14].

Thus, in the face of this varying band-edge, a conducting carrier faces a complex rolling landscape of “hills” and “valleys” and is imagined to progress through this landscape in a percolative manner. In addition to this, there is known to exist a set of bound states with energies below the band-edge energy that form so-called tail-states. This reality then motivates the origin of the term “mobility edge” as being the cutoff energy in the middle of a spectrum of states that separates the immobile, bound states for all energies below the edge from the free, conducting states at energies above it. The ultimate importance to transport modeling of these tail-states will be the topic of Section 2.4.

Given this microscopic picture of rolling band-edge hills and valleys, the current state-of-affairs wherein “compact” modeling is the central focus is somewhat unfortunate. Capturing the effect of percolation and three-dimensional spatial variation within an effectively zero-dimensional “compact” model is very difficult and demands a heavy amount of physical assumptions and approximations. For example, often the results of bond percolation theory [15,16] are invoked in such models [11,12,13,16], but the transport behavior of a saddle point between two valleys of a 3D-varying-electrical potential is not as simple as that of a single-valued resistor. Yet this is precisely what most bond percolation math assumes: reducing the problem to a randomized network of resistors. Furthermore, the results of such percolation theory analyses often require one to assume a certain geometry of connections in this effective-resistor-network (e.g., square lattice, hexagonal, etc.), and the results can only be considered valid in the limit of an infinitely large device.

However, in 3D TCAD, one does not have to rely on any of these assumptions or approximations. One can simply make a 3D-varying band-edge. We dub such a TCAD model a “microscopic” model, and it represents a very natural approach to studying and modeling a-IGZO. Yet to our knowledge, such models are effectively absent from the literature.

Thus, in this work we detail the construction of such a model and demonstrate its clear utility in a number of situations. In Section 2.1, we show how to algorithmically generate a spatially varying band-edge with a physically reasonable set of spatial correlations and the correct statistics according to Equation (Section 1). In Section 2.2, we will also draw attention to the difficulty of modeling a wide-band-gap semiconductor such as a-IGZO using conventional TCAD solvers based on drift diffusion with normal double numerical precision and will highlight the great value of the alternative quasi-Fermi-level transport (QFT) [17] formalism to achieve better convergence and results, especially when temperature-dependent studies are done. Then, in Section 2.3 we will show that the microscopic model created here can accurately reproduce experimental results. After this, in Section 2.4, the question of band-tails, their effect on the mobility, and how they can be added to a simulation in a numerically efficient manner will be detailed. Finally, in Section 3.1 and Section 3.2, the paper will be concluded with two short studies of 3D effects in a-IGZO modeling. The goal of these sections is to stoke interest and demonstrate the utility of and need for such microscopic TCAD modeling efforts rather than to be comprehensive studies in and of themselves, which will be left to later works.

## 2. The TCAD Model

As has been said, to date, the modeling of both a-IGZO bulk films and a-IGZO devices has been somewhat limited to “compact” models, and there have been relatively few attempts at TCAD modeling [18,19]. Although there is a great variety of such models [3,5,9,10,11], they all center around two key aspects: (1) the role of electrostatic disorder of the conduction band or mobility band-edge and (2) the role of trapping bands or band tails. A discussion of the second aspect will be left to Section 2.4.

The meaning of electrostatic disorder has somewhat changed from some of the earliest modeling efforts by Kamiya, Nomura, Hosono, et al. [3,4,7]. In those early works, a-IGZO was imagined to have a certain uniform conduction band or mobility edge, $E_{b0}$, and above that band-edge floor, a series of randomized potential barriers arise, hindering transport. However, this early idea was refined in later models [10,11,12,13] to, instead, a notion of a material wherein the conduction/mobility band-edge itself varies everywhere space according to Equation (1).

The effect that this spatially varying mobility band-edge should have on the charge density is clear and, to our knowledge, is agreed upon by all models of this type to take the form:

(2) $$n\left(E_{F}\right)=\int_{-\infty }^{\infty }P\left(E_{b}\right)\left[\int_{-\infty }^{\infty }DOS\left(E-E_{b}\right)f_{FD}\left(E,E_{F}\right)dE\right]dE_{b}$$

where $DOS(E-E_{b})$ is a density-of-states function centered about the energy $E_{b}$, $f_{FD}\left(E,E_{F}\right)$ is the Fermi–Dirac probability distribution, and $E_{F}$ is the Fermi level. The central crux of such an expression is that the average effect of a spatially varying band can be determined by substituting an integral over all space with an ensemble integral over all band-edge values weighted by the fraction of the system volume with that band-edge.

However, where these many “compact” models differ is in how they treat the conductivity. There is general agreement that for mobile electrons (in contrast to the bound and localized states that will be discussed in Section 2.4), the effect of percolation is very important, with free carriers taking a circuitous, percolative path from one end of the system to the other. However, though there is, in general, conceptual agreement on this point, the specific functional forms and modeling techniques and assumptions vary wildly.

On this point, one can see the great value of a TCAD model to the conversation, as with a TCAD model, one can capture these physical effects in a direct way—one simply inserts a spatially varying potential with the correct properties into the simulation. Thus, no ad hoc assumptions or hand-wavy insertions of percolation results from resistive networks or effective media are needed. Instead, the microscopic TCAD model presented here is fundamentally agnostic to these often difficult-to-justify approximations.

**Figure 1:**
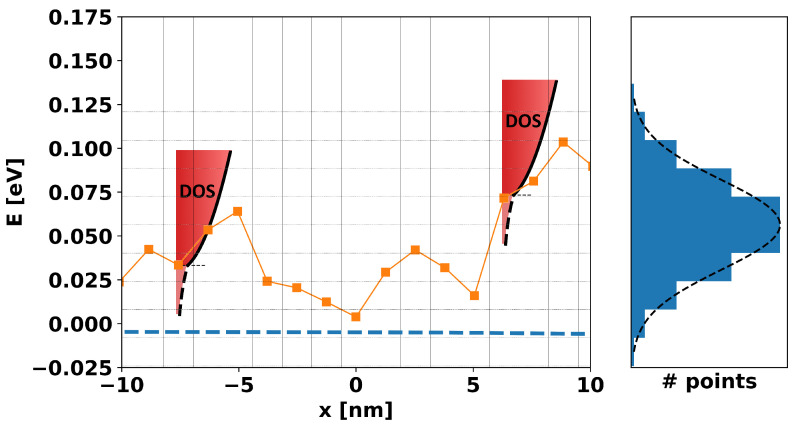
A schematic representation of the band-edge landscape and its concrete elements along a 1D cross-section. Gridding along the x-axis represents the Voronoi volumes associated with each mesh point along the cross-sectional line (squares), the orange line represents the band-edge at each point, and the dotted blue line is the Fermi level. The gridding along the y-axis represents energy ranges and corresponds to the histogram on the right showing the total number of mesh points with band-edges falling in the range between any given y-grid lines. Note that the histogram is representative of a much larger set of points than is shown in the figure, which is too small a sample for clear statistics. Finally, at any and all mesh points, we imagine a density-of-states (DOS) that includes the free and bound carriers centered around the band-edge energy at that mesh point.

However, in a real amorphous film, such rolling band-edge landscapes are not completely spatially random. Instead, based on microscopic details of chemical bond physics, strain, process effects, etc., there will be a certain characteristic size of “hill” and “valley” that should be replicated in TCAD simulation. Furthermore, even if this was not true, such spatial correlation is essential in order for any TCAD model to produce results that are deterministic and independent of grid-size and shape (provided that the grid is, in general, resolved finely enough to capture the spatial fluctuations). Thus, the first step in constructing a “microscopic” TCAD model (the key features of which are illustrated in Figure 1) is to understand how to generate a physically realistic band-edge landscape.

### 2.1. Correlated Gaussian Random Field Mobility Edges

The fundamental task at hand is to generate a spatially varying band-edge, $E_{b}\left(r\right)$, subject to the following two conditions:

1. Globally Gaussian distributed: Globally, the finite set of band-edge values, $\left\{{\left(E_{b}\right)}_{i}\right\}$, of the mesh, corresponding to certain values of the band-edge at each vertex *i* and integrated over the Voronoi or box volume associated with *i*, must follow the Gaussian distribution:
  (3) $$\frac{1}{\sigma_{E_{b}}\sqrt{2\pi }}\exp\left(-\frac{{\left({\left(E_{b}\right)}_{i}-\langle E_{b}\rangle \right)}^{2}}{2\sigma_{E_{b}}^{2}}\right)$$
  with standard deviation $\sigma_{E_{b}}$ and a mean of $\langle E_{b}\rangle$.
2. Locally spatially correlated: Band-edge values of nearby mesh points should not vary independently but, rather, exhibit a spatial (auto)correlation such that:
  (4) $$\langle {\left(E_{b}\right)}_{i}{\left(E_{b}\right)}_{j}\rangle =C(|r_{i}-r_{j}\left|\right)$$
  where $r_{i}$ represents the position of vertex *i*, and C(r) is a correlation function that must be given explicitly. By assuming that the correlation function only takes in a scalar variable representing the distance between points *i* and *j*, it is being assumed that the correlations are *homogeneous* and, thus, have no explicit spatial dependence (i.e., there are no “special” places and the correlations are the same everywhere) and are *isotropic* and, thus, have no explicit dependence on orientation or angle. The possible specific forms of the correlation function, C(r), are discussed in Appendix A.

**Figure 2:**
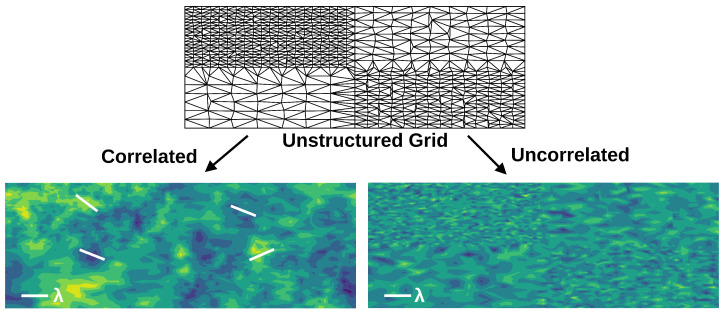
A schematic drawing of the field generated using a Gaussian random field (GRF) versus a completely uncorrelated field for an unstructured mesh constructed to have regions of noticeably different gridding. In the GRF, the characteristic size of fluctuations is agnostic to the gridding (provided the gridding is much finer than the correlation length), whereas for the uncorrelated result, it is visibly not agnostic to the gridding. This is further highlighted by the white lines in the correlated image that show some sample “hills” and “valleys” of a consistent size.

These combined properties are demonstrated in Figure 2.

Note that, in general, we would like an approach based on unstructured meshes, but the approach developed here relies on the use of Fourier transforms, which can be efficiently generated—via the use of the fast Fourier transform (FFT) algorithm—only on regular grids. One could attempt to construct an unstructured algorithm, but here we take the simpler approach of generating a field on a regular, finely meshed grid and using interpolation to map the values back to the unstructured one. To us, this approach is quite justifiable, as this band-edge generation step must only be done once at the beginning of the TCAD simulation and, thus, even though it relies on fine-gridding that is then potentially discarded, the total contribution to the numerical burden of the full simulation is typically minimal.

The first requirement stated above amounts to generating a so-called Gaussian random field (GRF), which is a well-known object in the field of statistics. The second requirement can be obtained by exploiting the Wiener–Khinchin (WK) theorem [20,21]. As both these aspects are well-studied topics in statistics, we will make no attempt to prove or justify them here. Instead, for completeness, in Appendix A, we provide a derivation of and the motivation for an algorithmic approach for correlated GRF generation, but in order to keep the main body of this manuscript concise, here we will only give the final resulting algorithm.

In summary, the procedure is as follows (shown for 3D, but the approach is also suitable for 2D):

1. Generate a field of $N_{x}\times N_{y}\times N_{z}$ values either according to Equation (A9) directly or through generating random Gaussian numbers of unit variance, N(μ,σ)=N(0,1), in real-space and performing a discrete Fourier transform of the values. We call the resulting field $\phi_{0}\left(k\right)$. (Input: $\phi_{0}\left(k\right)$ (if Equation (A9) used), $\phi_{0}\left(r\right)$; Output: $\phi_{0}\left(k\right)$).
2. Generate an $N_{x}\times N_{y}\times N_{z}$ grid of *k*-values, where $k=2\pi \sqrt{n_{x}^{2}+n_{y}^{2}+n_{z}^{2}}$, where $n_{i}$ has been *shifted* to include negative and positive values centered at k=0. Many numerical environments have built-in functions for this (for example, 2 * numpy.pi * numpy.fft.fftfreq((Nx,Ny,Nz)) in numpy). We call this grid |k| or simply *k* though it is three-dimensional. (Input:$N_{x}$, $N_{y}$ and $N_{z}$; Output: *k*).
3. Scale the field $\phi_{0}\left(k\right)$ by P(k), where P(k) is either Equation (A3) or (A4) (depending on the desired correlation) evaluated at each point *k*. This scaled field we call ${\hat{E}}_{b}\left(k\right)$. (Input: $\phi_{0}\left(k\right)$; Output: ${\hat{E}}_{b}\left(k\right)$).
4. Perform an inverse discrete Fourier transform of ${\hat{E}}_{b}\left(k\right)$ to yield $E_{b}\left(r\right)$. (Input: ${\hat{E}}_{b}\left(k\right)$; Output: $E_{b}\left(r\right)$).
5. Rescale and shift $E_{b}\left(r\right)$ according to Equation (A10) to give the final spatially correlated field. (Input: $E_{b}\left(r\right)$; Output: $E_{b}\left(r\right)$ (normalized)).

**Figure 3:**
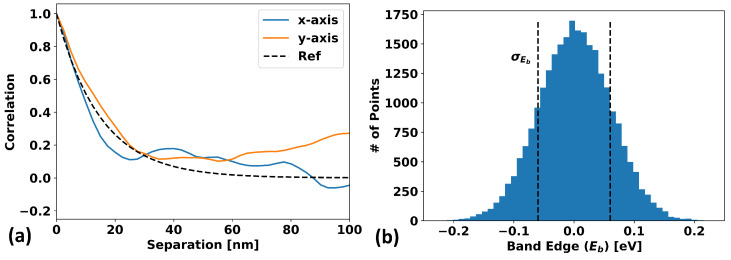
(**a**) The spatial correlation, $C\left(d\right)=\langle E_{b}\left(r\right)E_{b}\left(r+d\right)\rangle$, in the *x*- and *y*-directions (blue and orange, respectively) for a sample correlated Gaussian random field (GRF) constructed with exponentially decaying correlations (and a uniform grid of λ/3). (**b**) A histogram made of the $E_{b}$ values of every mesh point in the simulation, demonstrating that the GRF has the intended statistical distribution and standard deviation.

**Figure 4:**
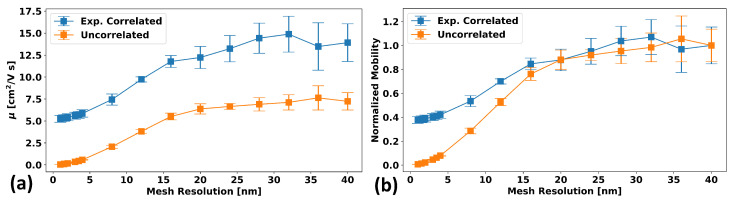
(**a**) The differences in the means and standard deviations of the calculated mobility for 10 different randomly generated GRF samples for exponentially correlated (blue) and uncorrelated (orange) spatially varying band-edges as a function of grid size for a fixed system size. It can be seen that a correlated model approaches saturation to a final finite value with minimal variation with sufficiently small gridding, whereas the uncorrelated approach saturates to zero mobility. (**b**) The same data as (**a**) but with mobilities normalized to their large grid size (i.e., right-most) value.

Once the above algorithm is performed (again, see Appendix A for a detailed derivation of this algorithm), the result is a field $E_{b}\left(r\right)$ with the desired properties. Figure 3 and Figure 4 demonstrate this for a sample GRF generated using this algorithm. Figure 3a shows that the generated GRF does indeed manifest the desired spatial correlations: in this case, exponentially decaying correlations with a correlation length of 20 nm are used, and the expected behavior is shown as a dotted black line. Figure 3b shows that the band-edge values at each vertex also follow the probability distribution dictated by Equation (1). Figure 4a considers the differences in the means and standard deviations of the calculated mobility for 10 different randomly generated GRF samples for the case of exponentially correlated and uncorrelated spatially varying band-edges as a function of grid size for a fixed system size. The model parameters and procedure used for the determination of the mobility are discussed in Section 2.3. It can be seen that a correlated model approaches saturation to a final finite value with minimal variation with sufficiently small gridding, whereas the uncorrelated approach saturates to zero mobility. Based on these results, it would seem that for mesh resolutions below approximately half the correlation length (10 nm here), the mobility reaches an acceptably saturated value with quite low variation. This implies that a mesh resolution of λ/2 or finer is desirable for simulation. Figure 4b is the same data as (a), only plotted with mobility values and standard deviations normalized to the large grid size (i.e., right-most) value.

### 2.2. The Quasi-Fermi Level Transport (QFT) Model

In our model, we assume drift diffusion (DD) transport through the spatially varying system. Furthermore, we assume a simple constant field- and temperature-independent mobility, $\mu_{0}$, at all mesh points. Note that, as will be seen in Section 2.3, this certainly does not mean that the a-IGZO layer as a whole will exhibit a constant field- and temperature-independent mobility. Rather, the spatially varying band-edge will inject complex transport physics driven by percolation effects into the transport characteristics of the device, and thus, the *emergent*, aggregate mobility of the whole device will indeed exhibit field and temperature dependence.

Furthermore, the assumptions of DD transport and a constant mobility are not essential ingredients to the model we present, and thus, other transport models and more complex mobility models can easily be used along with the spatially varying potentials of Section 2.1 and the band-tails that will be described in Section 2.4 to expand the basic model presented here. In fact, the use of a more complex mobility model with mobility degradation near the oxide surface will be considered later in Section 3.2.

However, even the use of the relatively straightforward and ubiquitous DD model requires special care when it comes to a-IGZO. In general, TCAD simulation in situations where carrier concentrations are very low presents challenges for numerical convergence. This is true for wide-band semiconductors such as a-IGZO and for systems at cryogenic temperatures where there are only very few thermally excited carriers. This issue is then especially bad if one wants to consider a wide-band-gap semiconductor like a-IGZO *at* cryogenic temperatures. Yet the study of the mobility of a-IGZO as a function of temperature is frequently a focus of experimental studies and thus of great interest for supportive TCAD simulations.

There are two main reasons for the numerical difficulties associated with lower carrier densities, and they can be understood by considering the fundamental current equation of the drift diffusion model:

(5) $$\frac{\vec{J}\left(r\right)}{q\mu_{0}}=-n\left(r\right)\nabla E_{b}\left(r\right)-k_{B}T\nabla n\left(r\right)$$

where $\vec{J}\left(r\right)$ is the current, $E_{b}\left(r\right)$ is the spatially varying band-edge, *q* is the electron charge, $k_{B}$ is the Boltzmann constant, and *T* is the temperature. The value n(r) is the carrier concentration, which may, in principle, be calculated using either Maxwell–Boltzmann or—as is more appropriate for a-IGZO, where the Fermi level frequently enters the conduction band—Fermi–Dirac statistics. However, in reality, in the usual implementation of drift diffusion, by using the so-called Scharfetter–Gummel (SG) scheme [22], Boltzmann statistics become explicitly baked-in to the model.

The first issue is what has been called *catastrophic cancellation* [17], where the first drift term and the second diffusion term are comparatively large but very close in absolute value such that they agree for many decimal places and only differ in the deep trailing decimal values. This creates a problem as the mantissa (i.e., decimal portion) of the double variables frequently used in numerical computation may not be sufficient to capture this finite, non-zero difference in nearly identical numbers as distinct from zero.

This first problem can be somewhat addressed by using a larger numerical datatype, such as a long double, and that is what is done in our model as well. However, there is an even greater issue, and that is that at low carrier concentrations, as the carrier concentration exponentially depends on the band-edge and/or Fermi level in a form $\exp\left(\left(E_{b}\left(r\right)-E_{F}\right)/k_{B}T\right)$, as *T* becomes small or $\left(E_{b}-E_{F}\right)$ becomes large, small changes in $E_{b}\left(r\right)$ can lead to dramatic changes in carrier concentration. This creates a great problem for meshing in TCAD, as all carrier concentration gradients will become sharper and sharper and more abrupt and thus require a finer and finer mesh in order to be spatially resolved as the temperature becomes lower or the band-gap becomes wider. Such ultra-fine meshes are obviously numerically cumbersome.

This issue can be greatly alleviated by reformulating the basic drift diffusion equation into a form we call “Quasi-Fermi-Level Transport” or QFT. This approach is expanded upon in greater detail in a previous publication by some of the authors [17] and so will not be fully described here. However, the salient idea of the QFT approach is to make the spatially varying quasi-Fermi level, $E_{F}\left(r\right)$, the key unknown quantity to solve for:

(6) $$\frac{\vec{J}\left(r,E_{F}\right)}{q\mu_{0}}=n\left(r,E_{F}\left(r\right)\right)\nabla E_{F}\left(r\right)$$

This is in contrast to the regular DD Equation (5), where the carrier concentration, $n(r,E_{F}\left(r\right))$, and the band-edge (or electrical potential), $E_{b}\left(r\right)$, are the unknown quantities. In DD, as there are two different quantities, one generally solves the equation iteratively until self-consistency is obtained between $n(r,E_{F}\left(r\right))$ and $E_{b}\left(r\right)$. However, in QFT, where the Fermi level is the solved quantity, this self-consistency is traded in for the basic equation being explicitly non-linear and with the coefficient of the gradient term being some complex function of $E_{F}$ (i.e., the Maxwell–Boltzmann or Fermi–Dirac equation weighted by some density of states).

There is also the subtle detail of how the current should be evaluated between mesh points. For DD simulation, as was said, the well-known Scharfetter–Gummel scheme is used and attempts to formulate the relevant current equations in a way that explicitly considers the exponential relationship between carrier concentration and potential, as assumed in Boltzmann statistics, in order to yield greatly enhanced numerical convergence. A similar scheme is also necessary in QFT, but luckily, such a scheme can be created with only small changes to the regular SG and can be extended to permit Fermi–Dirac statistics. Details about this and any further details about the properties of QFT are given in [17].

**Figure 5:**
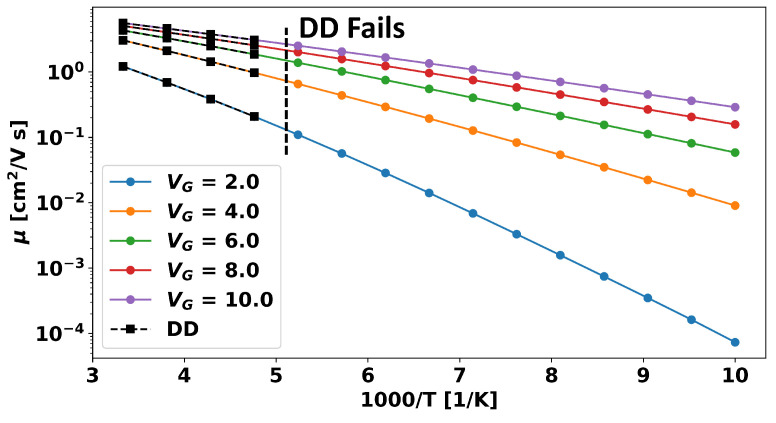
Mobility versus inverse temperature for different gate voltages with the parameters in Table 1 for a drift diffusion (DD)-based simulation versus one using quasi-Fermi-level transport (QFT). It can be clearly seen that for temperatures below ∼200 K, the DD-based solver fails to converge, and no value can be obtained; but the QFT approach yields sensible results. It can also be seen that both approaches produce identical results at higher temperatures.

Thus, our a-IGZO model assumes drift diffusion transport but casts the key equations in the QFT form. This, combined with the use of long double precision, allows the model to achieve much better convergence than regular DD. This is demonstrated in Figure 5, where the mobility (using the model parameters derived later in Section 2.3 and given in Table 1) is shown as a function of the inverse temperature for DD and QFT. As can be seen, at temperatures less than ∼200 K, the DD simulation fails to converge, whereas convergence can be obtained with QFT down to 100 K. Below 100 K, however, even the QFT results become increasingly noisy for the mesh size used (not shown). This could be improved by considering a higher mesh resolution, but experimental results on a-IGZO in the literature rarely go below 100 K, so we consider that limit to be sufficient for our purposes here.

### 2.3. Validation against Experimental Results

In order to demonstrate the physical accuracy of the TCAD model developed here, it must be validated against experimental results. Although there are a great number of experimental results available, here we make use of those from Germs et al. [5], which have already previously been used in the literature for model validation: both in the original paper itself and in [11].

**Figure 6:**
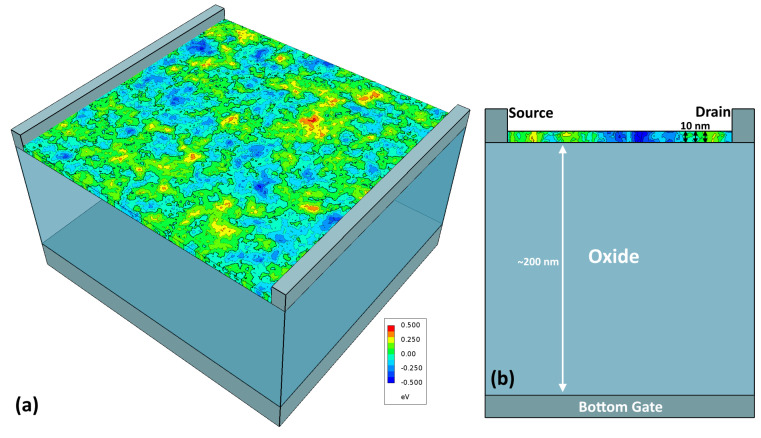
(**a**) A 3D diagram of the device simulated in Section 2 showing the spatially varying band-edge of the a-IGZO film generated using the procedure described in Section 2.1. (**b**) A 2D cross-section of the same device showing a 200 nm-thick oxide layer ($t_{\mathrm{ox}}$) and a 10-nm thick a-IGZO layer ($t_{\mathrm{IGZO}}$) on a back-gated device with Schottky/undoped source and drain contacts.

As described in Germs et al. [5], the measured device consists of a 10 nm-thick layer of a-IGZO atop a ∼200 nm-thick layer of SiO_2_, making for a back-gated device. In the process of fitting, the value of the a-IGZO layer was held fixed, but we found it beneficial to allow for the value of the oxide thickness, $t_{\mathrm{ox}}$, to vary by some percent and to thus act as a strongly constrained fittable parameter. Alternatively, this allowed for small variations to be considered to encapsulate any intermixing of the a-IGZO and oxide to create a thin intermediate layer of different permittivity. The a-IGZO film was then contacted on both sides by 25 nm-thick gold contacts with 5 nm-thick titanium adhesion layers—though within the TCAD simulation, they are simply treated as perfect conductors (note that this means that they have zero-resistance and is independent of whether they contact the channel in an Ohmic or Schottky way). The experimental devices had lengths and widths in the 100s of micrometers; however, it is both unnecessary and computationally prohibitive to simulate such large films while maintaining microscopic detail at the scale of 10s of nanometers. Therefore, the simulated device has a much smaller length and width of 200 nm, with the grid resolved to a scale of 2.5 nm. Numerical experimentation shows (as will be shown in Section 3.1) that this reduced device size has a negligible effect on the results provided that the device is still many times larger than the correlation length of the spatial fluctuations. A 2D cross-section of the final simulated device is shown in Figure 6b, though, note that as percolation physics plays a central role in transport, a 2D device has fundamentally different percolative behavior than a 3D device, and all simulations are thus 3D. The question of whether an effective 2D or “compact” model can be adapted from this 3D model is considered to be beyond the scope of this publication.

In order to fit the data, there are a number of parameters that must be set. The first issue is that a-IGZO devices such as those in Germ et al. [5] have no doped *n*- or *p*-junctions and, thus, are fundamentally junction-less or Schottky-based devices. Thus, Schottky contacts must be assumed to replicate accurate behavior, with the workfunction differences between the source (S) and drain (D)—which are assumed to have the same workfunction as they are the same material—and the a-IGZO channel (C) potentially playing an important role. We notate this value as $\Delta \Phi_{\mathrm{SC}}$, and the case for which it is zero corresponds to perfect ideal Ohmic contacts.

Assigning a single authoritative value to $\Delta \Phi_{\mathrm{SC}}$ is very tricky for a number of reasons. The first of these is that in the literature, both the values of the band gap, $E_{G}$, and the electron affinity, χ, of a-IGZO [23,24] fluctuate quite a bit, with typical values ranging from 3.0–3.5 eV and 4.0–4.5 eV, respectively. This uncertainty is compounded by the fact that the workfunction of the gold contacts, which is typically in the neighborhood of ∼5.0 eV, varies significantly depending on the process and Fermi-level pinning.

However, a greater issue is that even in the absence of Fermi pinning much of the “textbook” intuition around the expected band bending in a Schottky contact that is observed in conventional semiconductors like silicon does not apply in ultra-low-carrier-concentration materials like a-IGZO. For example, conventionally, one has a Schottky contact boundary dictated by the difference between the work function and the electron affinity of the metal and semiconductor, respectively, which then transitions as one moves deeper into the semiconductor to bulk semiconductor behavior. However, in a-IGZO, one will never observe bulk behavior.

The reason for this is because the length-scale of this transition from a Schottky boundary to bulk behavior is related to the Debye length of the semiconductor, which is based not only on the permittivity (here, a permittivity, $\varepsilon_{\mathrm{IGZO}}$, of 10.0 was assumed) but also on $\propto 1/\sqrt{n}$, where *n* is the electron concentration. In undoped a-IGZO with a band-gap of 3.2 eV—and, thus, an intrinsic carrier concentration that is $\approx 10^{18}$ times less than that of silicon—this Debye length at room temperature may actually be in the range of kilometers. Therefore, the difference between the average band-edge and the Fermi level everywhere in the device is entirely determined by the contacts in a-IGZO: the channel is extremely electrostatically flat (when neglecting the band-edge fluctuations), and any Schottky barrier mainly represents an “effective” value resulting from the competing electrostatics of the gate and source/drain contacts.

However, perhaps the biggest issue with assigning a definitive value to $\Delta \Phi_{\mathrm{SC}}$ is the fact that the “doping concentration” of a-IGZO can be a somewhat nebulous concept: a-IGZO is generally not doped with conventional dopants but, rather, the oxygen vacancies present within the film as a result of fabrication processes dictate the unbiased intrinsic Fermi level. Furthermore, for an “undoped” a-IGZO film, even a small concentration of the dopant-behaving vacancies can cause a substantial upward-in-energy movement of the Fermi level towards the conduction band-edge due to the ultra-low intrinsic carrier concentration. Thus, the “intrinsic” Fermi level of the experimental film cannot reasonably be expected to actually be in the mid-gap if any oxygen vacancies may be present, and this will manifest as a reduction in $\Delta \Phi_{\mathrm{SC}}$ as the average band-edge is pulled down (i.e., the Fermi level goes up due to dopant-behaving vacancies).

Thus, we assumed a fixed band gap value of 3.2 eV, and in light of all these considerations, we treat the contact workfunction $\Delta \Phi_{\mathrm{SC}}$ as a fittable parameter encapsulating the physics of both the doping level and the material energetic difference and allow it to take a fairly broad range. Furthermore, due to the wide band gap and the system always only operating in the n-type regime, the simulation of holes was neglected.

In addition to the contact workfunctions, $\Delta \Phi_{\mathrm{SC}}$, a value for the workfunction difference between the doped-Si bottom gate (G) and the a-IGZO channel and/or source/drain contacts must be set, which will determine the threshold voltage, $V_{\mathrm{th}}$, of the “turn-on” of the device. Here, we choose to denote this quantity as $\Delta \Phi_{\mathrm{GS}}$ and define it as the offset relative to the source/drain contacts rather than defining it relative to the channel due to the extremely weak electrostatic influence of the channel itself and the lack of any identifiable “bulk” region being present anywhere in the device. As before, this $\Delta \Phi_{\mathrm{GS}}$ is simply treated as a fittable parameter, and, unlike the Schottky contact workfunction value, we can expect $\Delta \Phi_{\mathrm{GS}}$ to potentially vary wildly in value depending on the number of traps present at both the gate and the a-IGZO sides and based on other process conditions.

**Figure 7:**
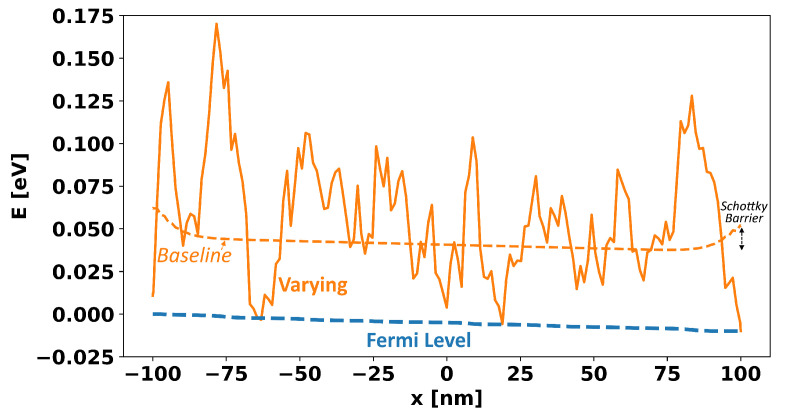
A plot of the band-edge versus position (i.e., $E_{b}\left(r\right)$) along a line through the center of the film along the width and thickness axes and spanning the length of the film from the source to the drain contact. The original baseline potential is shown as a dotted orange line representing only the effect of the Schottky contacts and gate workfunction, and the final simulated potential is shown as a solid orange line after the randomly generated band-edge variations were added. The Fermi level is also shown with an applied $V_{D}$ of 0.01 and the reference level of the energy being set by the left-most Fermi level.

As a result of these workfunctions, there is a certain non-uniform *baseline* band-edge reflecting only the Schottky contacts and gate workfunction, onto which the additional Gaussian band-edge variation is added. A 1D cross-section from the source to the drain of these two band-edges is shown in Figure 7.

As the mobility being modeled is an effective mobility for the entire system as controlled by the percolative impediment a carrier faces as it traverses the system, assigning a single-valued mobility to the system cannot be done by doing some sort of integral of the local mobility at all points. Rather, here we define the final TCAD mobility, μ, in a manner directly identical to how it is extracted from actual experimental measurements on FET devices as:

(7) $$\mu =\frac{L}{W}\times \frac{1}{V_{D}C_{\mathrm{ox}}}\times \frac{dI_{D}}{dV_{G}}$$

where *L* is the system length and *W* is its width (here, in both cases, 200 nm), and $C_{\mathrm{ox}}=\varepsilon_{0}\varepsilon_{\mathrm{ox}}/t_{\mathrm{ox}}$ is the capacitance of the oxide layer. $I_{D}$ and $V_{D}$ are the drain current and bias, respectively (in all simulations, a $V_{D}$ of 0.01 V was used), and $V_{G}$ is the gate voltage, which varied from 0 V to 20 V.

Finally, with the geometry- and device-specific parameters set, what remains is to determine the microscopic model properties of the a-IGZO film itself. These include: the correlation length of the band-edge variation, λ; the standard deviation of the band-edge variation, $\sigma_{E_{b}}$; and the constant bulk mobility, $\mu_{0}$. The average value of the band-edge, $\langle E_{b}\rangle$, is not treated as a fittable parameter but is instead dictated by the band gap, which, as mentioned previously, is given the fixed value of 3.2 eV.

Although the correlation length can be treated as a fittable parameter, instead, we fix its value to be 10 nm. This corresponds to the typical size of a spatially varying band “hill” or “valley” of ∼20 nm in diameter (i.e., the correlation length effectively defines the radius of influence about a given point). We believe this to be a reasonable value that is justifiable by, for example, some scanning tunneling microscopy (SPM) measurements [25] on a-IGZO films, where characteristic structures of roughly that size were found. However, we also admit that studies that reveal such information are rare and can often vary wildly in their observed fluctuation size, and thus, it may be necessary to treat λ as a fittable parameter in general.

**Figure 8:**
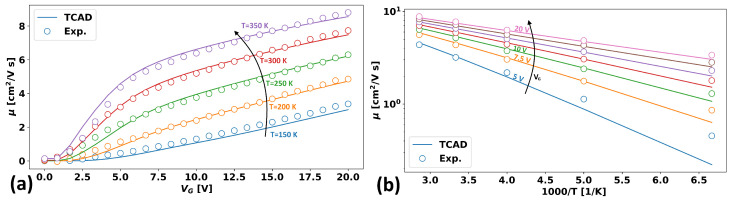
(**a**) TCAD versus experimental results from Germs et al. [5] for the mobility as a function of voltage for different temperatures from 150 K to 350 K. TCAD fits were done using the parameters in Table 1. (**b**) An alternative representation of the same data as in Figure (**a**) plotted instead as a function of the inverse temperature for a fixed gate voltage, $V_{G}$.

Thus, the set of fixed values is $t_{\mathrm{IGZO}}$, $E_{G}$, $N_{D}$, and λ, and the set of fittable parameters is $t_{\mathrm{ox}}$, $\Delta \Phi_{\mathrm{SC}}$, $\Delta \Phi_{\mathrm{GS}}$, $\sigma_{E_{b}}$, and $\mu_{0}$. Experimental reference values were extracted directly from Figure 4 of [5], and an optimization algorithm was run to optimize the TCAD model to match that data set. The final optimal values are listed in Table 1, and the resulting fits are shown in Figure 8a,b, which are the same data values plotted with different x-axes in order to highlight the gate voltage dependence and the temperature dependence of the mobility, respectively.

As can be seen, the TCAD model reproduces the experimental results very nicely for almost all the range of gate voltages (except, perhaps, the subthreshold region) and much of the temperature range (with some deviation at low temperatures for, again, the subthreshold region). However, the model considers none of the typical mechanisms for subthreshold degradation, such as interface traps—though such mechanisms could easily be added—and thus, the discrepancy is perhaps not surprising.

As has also been found in previous modeling attempts in the literature [10,11], an impressive fit to experimental mobility results can be obtained without considering the effect of bound band-tail states below the mobility band-edge. However, the existence of such band-tails are still a known reality of a-IGZO films, and thus, we now turn to the question of their inclusion in our microscopic TCAD model.

### 2.4. Inclusion of Band-Tails and Hypergeometric Functions

As was originally shown by Sir Nevill Mott in a seminal 1967 work, in the presence of heavy disorder—such as in non-crystalline, amorphous, impurity-heavy, or degenerately doped systems—under a fairly generic set of assumptions, the energy states of a physical system will decompose into two distinct energy ranges: a spectrum of non-localized mobile “free” states for energy above a certain cut-off value and a spectrum of non-conducting localized trap-like states for energies below that value [26,27]. This specific value that separates these two non-conducting and conducting spectral regions is then called the “mobility edge”. These states, like all trapping states, mean that only some fraction of electrons that comprise the charge density of a disordered system actually contribute to its conductivity.

These so-called “tail states” or “band-tails” of bound states that hang below the mobility band-edge are usually assumed to either have a Gaussian or exponentially decaying energy dependence for their density of states. In a-IGZO, the presence of these bands has been measured [28,29,30,31] and has been found to be mainly of the exponentially decaying type with a density of states (DOS) given by:

(8) $${\mathrm{DOS}}_{bound}\left(r,E\right)=\sum_{i}N_{m,i}\exp\left(\frac{E-E_{i}\left(r\right)}{k_{B}T_{0,i}}\right),\;E<E_{i}\left(r\right)$$

The sum represents the potential to have multiple exponential band-tails with a density-of-states constant of $N_{m,i}$, starting at an energy $E_{i}$, and having a characteristic decay length of energy of $k_{B}T_{0,i}$. The value of $E_{i}$ is defined relative to the band-edge $E_{b}\left(r\right)$ and, thus, spatially varies up and down throughout the system in a manner directly following the band-edge. Thus, to make this clear, we explicitly notate it as position-dependent. Alternatively, one can conceptualize $T_{0,i}$ as the system temperature above which carriers can free themselves from the bound trap states to populate the mobile, delocalized states. Most experiments [28,29,30,31] show that a maximum of two bands may be present (assuming deep traps are neglected) but that often only a single band is necessary to adequately match experimental results.

Given these results, we specialize our interest here to the case of only a single band-tail of the exponentially decaying type, though the inclusion of Gaussian tails is similar, although the integrals involved cannot be directly analytically solved—as they will be in Section 2.4.1 for the exponentially decaying case—and require either numerical integration or an analytical approximation, such as in [32].

However, despite the existence of these band-tails in a-IGZO being beyond dispute, their relative importance in the modeling of the mobility in a-IGZO is somewhat contentious in comparison to that of other amorphous materials such as a-Si, where their effect is known to be crucial. Some theoretical studies have argued that their inclusion is necessary to match experimental results [5]. However, often such studies treat the effect of the conduction band variation explored in Section 2.1 using imperfect models or neglect it entirely and, thus, may need to anomalously enhance the effect of these bands to unphysical levels in order to compensate for this important omission. Conversely, the majority of modeling works that demonstrate experimental matching [4,10,11]—including our own in the previous section—only pay lip service to the presence of such tails but then neglect them entirely when actually performing parameter fitting. This strongly suggests that their role is perturbative at best.

There is also a matter of dispute regarding how mobile these “bound” states should be. Many models [4,10,11] assume that electrons in these states are entirely immobile and can only contribute to conduction through the process of so-called “multiple catch and release”, where they thermally excite upwards in energy to mobile states, drift a bit, then are recaptured. However, others allow for so-called variable-range hopping (VRH)—a concept and model also introduced by Mott [33]—from trap-state to trap-state.

Thus, depending on the modeling objectives, it may be necessary to include multiple tails, with the carrier densities within these tails following their own mobility models. We now demonstrate how such tails can be integrated into the TCAD model considered here.

#### 2.4.1. Analytical Evaluation of the Band-Tail Charge Density

In principle, the inclusion of exponential band-tails in a simulation is straightforward if numerical integration is performed for the relevant integral:

(9) $$n_{bound}\left(r,E_{F},T\right)=\sum_{i}\int_{-\infty }^{E_{i}\left(r\right)}{\mathrm{DOS}}_{bound}\left(r,E\right)/\left(1+\exp\left(\frac{E-E_{F}}{k_{B}T}\right)\right)dE$$

However, in a TCAD simulation, this numerical integration would need to be performed at each and every mesh point, which would dramatically increase the numerical burden. However, for the specific case of exponentially decaying band-tails using the Fermi–Dirac distribution, an analytical evaluation is possible by recasting Equation (9) as:

(10) nbound(r,EF,T)=∑iNm,i(kBT0,i)×F121,1α,1α+1,−expEi(r)−EFkBT0,iα,α=kBT0,ikBT

where

(11) F12(a,b,c,x)=Γ(c)Γ(b)Γ(c−b)∫01tb−1(1−t)c−b−1(1−xt)−adt

is a so-called *Gaussian hypergeometric function*, with Γ(x) being the Gamma function.

Although this mathematical equality has been identified and exploited in many places in the literature [34,35], to our knowledge, this unexpected and somewhat esoteric equivalence has not actually been proven within the literature and has instead been simply stated without derivation. Thus, for conceptual clarity and convenience, in Appendix B, we provide a proof of this equality.

The great value of this connection with Gaussian hypergeometric functions is that such functions are well-studied and have many known useful analytical properties. For example, their derivative

(12) ddxF12(a,b,c,x)=abcF12(a+1,b+1,c+1,x)

is also analytically known, which can be quite useful when, for example, one is using a Newton simulation loop, which benefits from knowing the derivative of the charge density with respect to the potential and/or Fermi level.

However, an even greater benefit for recasting the key integral in terms of a Gaussian hypergeometric function is that a number of common numerical libraries have dedicated, numerically efficient functions for their evaluation. For example, in Python, there is scipy.special.hyp2f1, and in Boost C++, there is hypergeometric.hpp. In fact, although in the literature there have been a number of *approximations* to this integral developed [35,36,37,38] for use in, for example, compact circuit models, for the purposes of TCAD, we find that any form of approximation is completely unnecessary, as when one uses such pre-existing libraries, the evaluation of these integrals on every mesh point only contributes a tiny fraction to the overall simulation time.

**Figure 9:**
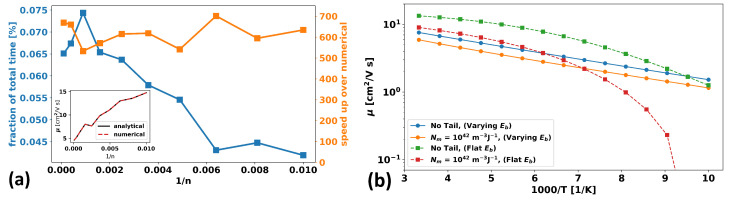
(**a**) Calculation time comparison of analytically evaluated vs. numerically integrated band-tail charge densities as a function of the inverse of the number of mesh points, *n*, in the device mesh. The left y-axis shows the percent of total simulation time dedicated to the analytical calculation (blue), and the right y-axis shows the speed-up of analytical evaluation compared to numerical integration (orange). The inset shows a negligible difference in the output mobility values for both methods as a function of system size. (**b**) Mobility as a function of inverse temperature for cases with (orange and red) and without (blue and green) band-tails and cases with (orange and blue) and without (red and green) a spatially varying band-edge.

To demonstrate this relative numerical lightweightness, we consider the case of a single trap band with a $T_{0}$ of 770 K and a density-of-states constant of $N_{m}$ of $10^{42}$ $m^{-3}$$J^{-1}$ (1.6 $\times \;10^{17}$ ${\mathrm{cm}}^{-3}$eV${\mathrm{eV}}^{-1}$). We simulate the device from Section 2.3, but we vary the mesh resolution, thus increasing the number of mesh points, *n*, and, thus, the number of band-tail integrals that must be evaluated. We track the computation time required for only the trap-band evaluation portion of the simulation as well as the discrepancy in value between a numerical integration versus an analytical evaluation of the band-tails using scipy.special.hyp2f1. The results are shown in Figure 9a. The left y-axis shows the percent of the total simulation time that is spent evaluating the analytical Gaussian hypergeometric functions. Never does this exceed 0.1% of the total simulation time. Furthermore, the right y-axis shows the relative speed-up of analytical evaluation over numerical evaluation, which is about a 650× speed-up for all system sizes. Finally, the inset shows that there is no detectable difference in the computed mobility values whether numerical or analytical approaches are used.

#### 2.4.2. The Effect of Band-Tails on Mobility

Figure 9b shows how the mobility varies as a function of the inverse temperature at a high gate bias of 20 V in the case where there is no trapping band present versus the case where there is one with a density-of-states constant $N_{m}$ of $10^{42}$ m$m^{-3}$ $J^{-1}$ (1.6 $\times \;10^{17}$ ${\mathrm{cm}}^{-3}$ ${\mathrm{eV}}^{-1}$) and a $T_{0}$ of 770 K. Additionally, the difference between a varying band-edge (using the parameters from Section 2.3) versus a flat non-a-IGZO-like band-edge are shown. It is immediately clear that the presence of a varying band-edge significantly changes the quantitative and qualitative behavior of the temperature dependence of the mobility.

When there is no varying band-edge landscape, the effect of traps is to substantially erode the mobility at low temperatures as more and more carriers freeze into the non-conducting tail-states. However, when varying band-edges are present, the behavior is quite different. The mobility is eroded somewhat since some carriers now lie in the band-tail and thus do not contribute to the conductivity, but this seems to yield a constant shift that is effectively temperature-independent.

**Figure 10:**
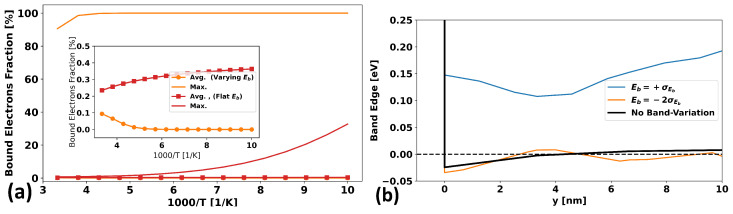
(**a**) The average fraction (with symbols) and maximum fraction (no symbols) of bound carriers (i.e., the ratio of the bound carrier concentration to the total carrier concentration) as a function of the inverse temperature when the entire 10 nm film is considered for the cases of a varying band-edge (orange) and an unvarying band-edge (red). (**b**) The band-edge as a function of position along the axis normal to the semiconductor–insulator interface for the cases of an unvarying potential and a varying potential, where two representative points have been chosen near the center of the device such that the mesh point right at the interface has a value of one standard deviation above the average (blue) or two standard deviations below the average (orange), thus representing a typical hill and deep valley, respectively. The Fermi level is also shown.

This difference in behavior is shown to be even more unusual when looking at Figure 10a, where the average and maximum fractions of bound electrons (averaged over the full 10 nm film) are shown for the two cases in question. It seems that for the varying-band case, the average fraction of bound carriers actually goes to zero at low temperatures, while the maximum fraction goes to 100%. This is in great contrast to the case of an unvarying band-edge, where the average fraction of bound carriers increases with decreasing temperature.

This is because, whether the band-edge is varying or not, the electrostatic effect of the gate contact forces the system to produce a certain amount of charge. However, when the band-edge is varying in space, there is still only a single Fermi level across the whole system. When the band-edge is not varying, the system is forced to form a surface charge layer that is effectively uniform throughout the device (neglecting the effect of the Schottky contacts), but when it is varying, most of the charge required by electrostatics is given by the “valley” regions—i.e., those regions where the band-edge is lowest—whereas the “hill” regions of high $E_{b}-E_{F}$ contribute very little.

Thus, the overwhelming majority of the charge required to compensate for the gate electrostatics comes from the comparatively small volume fraction of the films with deep valleys, and in these valleys, most carriers are free, leading to the fraction of bound carriers being very small. This can be seen by considering a 1D cross-section of the film at two points—one where the band-edge is quite low (i.e., a “valley”) and one where it is quite high (a “hill”)—and comparing this to the case where the band-edge is not varying at all. This cross-section is shown in Figure 10b for two specific points in the plane of the film that were chosen to be near the center (in order to minimize the effect of the contacts) but to have a band-edge of one standard deviation above the average band-edge (i.e., $+\sigma_{E_{B}}$, a “typical” hill) at the exact semiconductor–insulator interface and that of a very low valley with a band-edge of two standard deviations below the average band-edge value (i.e., $-2\sigma_{E_{B}}$, a “deep” valley) at the interface.

As can be seen, the low valley is below the Fermi level and lower than the unvarying case for most of the film thickness, with the Fermi level being deep in the band. Thus, the overwhelming majority of carriers are free carriers.

Capturing effects like this is very difficult in a “compact” model and even in TCAD models that use only an effective uniform mobility model. Thus, we see the great value of using an explicitly microscopic TCAD model when considering trapping-band behavior.

## 3. Model Application

In this final section of the paper, we briefly apply the developed TCAD model to two situations that highlight even further the great value of a microscopically accurate representation for capturing spatial effects in a very natural way that would be very difficult to incorporate in a non-TCAD model. We stress that the purpose of these brief studies is to promote the value of such a model rather than intending to represent a comprehensive study of each topic. Thus, each study is, by design, only superficial and is intended to only scratch the surface of a topic that begs further exploration.

### 3.1. Correlation Length Size Variation

A key parameter of the microscopic TCAD model presented here is the spatial correlation length, which dictates the characteristic sizes of “hills” and “valleys” in the varying band-edge landscape. As was discussed in Section 2.3, this value can be estimated based on experimental measurements. However, mobility modeling in a-IGZO is usually cast in terms of percolation theory [15,16], for which the analytical results are only valid in the limit of an infinitely large system.

Therefore, it is a natural question to ask what happens to the mobility in an a-IGZO film as its size approaches that of its spatial correlation length. More specifically, how does this affect the average value of the mobility and, also, its variability as one generates different random realizations of the correlated field?

**Figure 11:**
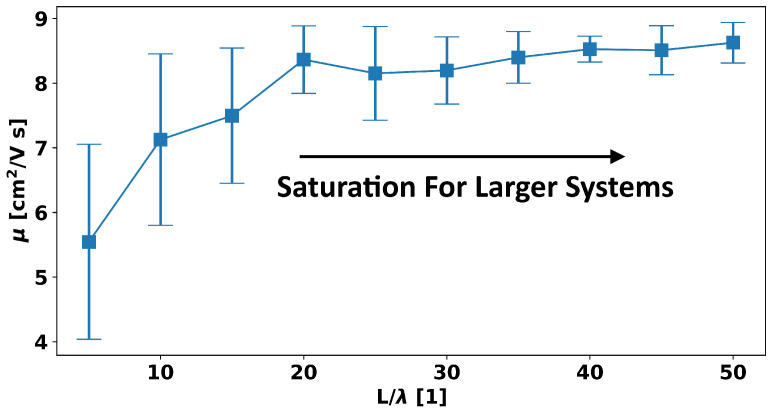
The means and standard deviations of device mobilities over 15 random samples of band-edge variational landscapes as a function of system length or width (a square film of fixed 10 nm thickness and a mesh size of 2.5 nm in the in-film direction and 1 nm in the out-of-film direction is assumed) divided by the correlation length.

Figure 11 assumes a device with the parameters from Section 2.3 of an unchanging 10 nm thickness and fixed grid size of 2.5 nm in the in-film direction and 1 nm in the out-of-film direction and that is square with an equal length and width (i.e., it has a size of L×L), and we consider how the mobility changes as a function of this length, *L*. For each value of *L*, 15 different random band-edge landscapes are then generated using the same exponentially decaying correlations as in Section 2.1 and a spatial correlation length, λ, of 10 nm. The means and standard deviations of the mobilities of these 15 realizations for each system size are then plotted as a function of the ratio of the system size to the spatial correlation length.

As can be seen, the mobility effectively saturates for system sizes larger than 20λ, or 200 nm (which is why this size was used in Section 2.3), and the variation (i.e., standard deviation) becomes small. However, for systems smaller than this, the mobility decreases substantially—by as much as ∼55%—with the variability becoming extremely large, such that for a system of 5λ, the variation is as large as 50% of the total value.

The reason for this large variation is because in such small systems, for some random landscape realizations, there may be no percolative path with low resistance through the system at all, and in others, there is a large one; thus, in the former, the mobility is severely degraded, and in the latter, the mobility approaches near to the value of that of very large systems.

Thus, such studies demonstrate the great utility of using such a microscopic TCAD scheme for the design of smaller a-IGZO devices provided that the spatial correlation length used, which is likely thickness and process dependent, can be estimated from experiments.

### 3.2. Surface Roughness

As a second and final investigation, we consider the effect of eroded surface mobility on a-IGZO. The topic of surface roughness on a-IGZO is little-studied, and the studies that do exist [39] largely ignore the reality of a varying band-edge in their consideration. However, realistically, we expect this variation to have a non-negligible role as, effectively, there is now a second length-scale due to the range of surface roughness scattering, $\lambda_{sr}$, entering the physics (the first length-scale being the correlation length of spatial variation, λ).

To provide a first motivation for this statement, we modify the TCAD mobility model from one that is constant at all mesh points to one for which the mobility is reduced exponentially to a final surface value, $\mu_{surf}$, depending on the distance from the semiconductor–insulator interface, *y*. Thus, the mobility has the following form:

$$\mu \left(y\right)=\mu_{0}+\left(\mu_{surf}-\mu_{0}\right)\exp\left(-\frac{y}{\lambda_{sr}}\right)$$

**Figure 12:**
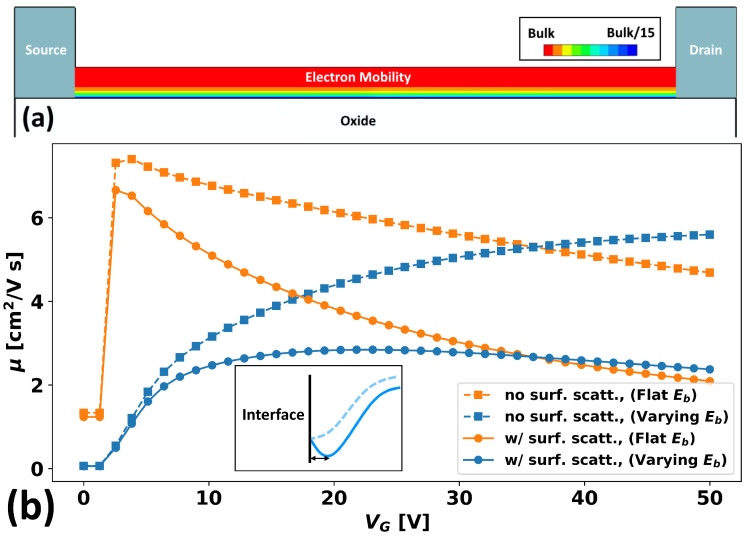
(**a**) A cross-sectional diagram showing the spatial variation in the mobility as a function of the distance from the semiconductor–insulator interface. (**b**) Mobility vs. $V_{G}$ for the cases of varying (blue) and unvarying band-edges (orange) for the cases of uniform mobility (dotted squares) and surface-reduced mobility (circles with solid lines) models. The inset depicts a proposed reason for the reduced surface scattering in the varying band-edge case, as the carrier may favor deeper valleys (darker blue, solid) that are further from the interface over shallow valleys (lighter blue, shallow) that are closer.

Figure 12 shows the mobilities for varying and unvarying band-edges in the presence or absence of surface roughness scattering as a function of gate voltage. As gate voltage increases, this will push the carriers closer and closer towards the interface and, thus, into the region of degraded mobility. We take $\mu_{0}$ to be the same as in Section 2.3, take the eroded surface value, $\mu_{surf}$, to be 1 ${\mathrm{cm}}^{2}$/V·s (approximately 1/15 of $\mu_{0}$), and take $\lambda_{sr}$ to be 1 nm.

Looking at the figure, for the case where the surface mobility is the same as the bulk mobility, we observe nearly voltage-independent mobility for the case of an unvarying band-edge, as expected. Any non-uniformity is due to the effects of the Schottky contacts. This is in contrast to the case of a varying band-edge, where we see that the mobility increases with the gate voltage, as in Figure 8. This is because in a-IGZO, unlike in silicon, mobility increases with carrier concentration, and thus, as more and more carriers are required and they are crammed into a thinner and thinner surface layer, mobility is enhanced. Alternatively, one can say that at high gate bias, the Fermi level is brought higher: usually resulting in the varying band-edge “filling up” the valleys to higher levels and thus opening more percolative paths through the landscape.

However, when surface scattering is then added, the behavior in both situations changes in different ways. For the non-varying band-edge, the mobility is continually degraded as $V_{G}$ is increased (up to at least the final $V_{G}$ of 50 V). Conversely, for the varying band-edge, mobility seems to saturate at a certain value that is higher than the lowest value observed for the unvarying case (i.e., the unvarying mobility drops below the varying mobility at ∼35 V). This is an unexpected finding and likely merits additional study. A plausible reason for this is that in the varying band-edge case, only carriers in the deep valleys of the landscape are contributing to the mobility, and they can afford to be farther from the interface if the minima of the valley sits some distance away from the interface. This is depicted pictorially in the inset of Figure 12, which highlights how carriers may favor a deeper valley, even if it is farther from the interface, over a shallower valley that is closer to the interface.

Thus, we see another case where a microscopic TCAD description provided important utility and insight that a compact or effective (i.e., spatially uniform) TCAD model would miss.

## 4. Conclusions

In this paper a so-called “microscopic” TCAD model of a-IGZO is presented. Key aspects of its implementation are detailed, and its ability to reproduce experimental results is demonstrated. The issue of the addition of non-conducting band-tail states in a numerically efficient manner is addressed. Finally, two short studies of 3D effects are undertaken to motivate the utility of such a TCAD model: specifically, the cases of variational effects as a function of device size and as a function of surface roughness scattering. Both of these studies demonstrate how subtle aspects of the 3D distribution of charge in an a-IGZO film, with its varying band-edge landscape, leads to results that are fundamentally different than the expectations from a more “compact” zero-dimensional model.

## Figures and Tables

**Table 1:** Optimal parameters found for fitting the experimental results of [5].

Fixed Parameters	Value
IGZO Thickness ($t_{\mathrm{IGZO}}$)	10 nm
IGZO Bandgap ($E_{G}$)	3.2 eV
IGZO Permittivity ($\varepsilon_{\mathrm{IGZO}}$)	10.0
IGZO n-Doping ($N_{D}$)	$10^{5}$ ${\mathrm{cm}}^{-3}$
Spatial Correlation Length (λ)	10 nm
**Fitted Parameters**	**Value**
Oxide Thickness ($t_{\mathrm{ox}}$)	218.5 nm
Source-Channel Workfunction ($\Delta \Phi_{\mathrm{SC}}$)	62 meV
Gate-Channel Workfunction ($\Delta \Phi_{\mathrm{GS}}$)	1.543 eV
Band-Edge Standard Deviation ($\sigma_{E_{b}}$)	112.5 meV
Bulk IGZO Mobility (i.e., if no variation) ($\mu_{0}$)	16.75 ${\mathrm{cm}}^{2}$/V s

## Data Availability

The raw data supporting the conclusions of this article will be made available by the authors on request.

## Appendix A. Generating A Gaussian Random Field with Desired Spatial Correlations

In this appendix, the algorithm for the generation of the spatially correlated Gaussian random field (GRF) mentioned in Section 2.1 is derived.

There are multiple equivalent ways of defining a GRF or the related concept of a Gaussian process (a Gaussian field being a mathematical object with a random value assigned to each of a finite set of points in space assigned according to a Gaussian process). One definition is to consider expanding any arbitrary statistical field in cumulants or moments—a similar and analogous procedure in the field of statistics to expanding a function in a power series—and to define a GRF as a field wherein all higher-order terms beyond the mean (first-order) and standard deviation (second-order) are zero. These higher-order terms represent properties such as skewness, which a Gaussian process does not have, and thus, an alternative statement of the definition of a GRF is a field for which its statistics are completely determined by its mean and standard deviation.

One way of stating the crucial central limit theorem of the field of statistics is that if one has a set of random numbers $\left\{X_{i}\right\}$, even if they themselves were not drawn from a Gaussian distribution, the recast variable $x_{i}=\sqrt{n}\left(X_{i}-\langle \{X_{i}\}\rangle \right)$*will* approach a Gaussian distribution with a standard deviation of the original variable set for a large enough number of samples *n*. Thus, there are many, many approaches by which one can generate a set of points with a Gaussian distribution, even without starting with Gaussian random number generation. However, the true difficulty is generating a GRF with the requisite correlations. For this, one can exploit the Wiener–Khinchin (WK) theorem.

Although the WK theorem can be stated in many highly generalized ways, for our purposes here, we can state it as:

(A1) $$P\left(|k|\right)=\int_{-\infty }^{\infty }C\left(r\right)e^{-i|k|r}dr,\;\left|k\right|=\frac{2\pi n}{N},\;n=0,1,2,\ldots ,N-1$$

This can be stated in words as: *the Fourier transform of the correlation function of a well-behaved statistical field (Gaussian or otherwise) is the power spectrum of that field.*

The power spectrum, P(|k|), is the squared amplitude of a field—in this case, $E_{b}\left(r\right)$—in reciprocal- or *k*-space:

(A2) $$P(|k|)=|{\hat{E}}_{b}(|k|)({\hat{E}}_{b}{\left(|k|)\right)}^{*}{|}^{2}=|{\hat{E}}_{b}{\left(|k|)\right|}^{2}$$

where ${\hat{E}}_{b}\left(|k|\right)$ is the Fourier-transformed (or discrete-Fourier-transformed (DFT), as the values of $E_{b}\left(r\right)$ are a finite set on a grid) values of the original field values. Note that by again invoking an assumption of homogeneity and isotropy, as was done in Section 2.1, the power spectrum depends only on the magnitude of k, and thus, from here on, we will simply write it as *k*.

This WK theorem property is very useful, due to its inverted form: if one can generate a GRF with a certain power spectrum, then that GRF will obey spatial correlations corresponding to the Fourier transform of the power spectrum used. In this work, we specialize our considerations to two types of spatial correlations: *exponentially decaying* and *Gaussian decaying*. To exploit the WK theorem, we must know both the functional form of the intended correlations in real-space and also their Fourier-transformed form (i.e., their associated power spectrum).

For exponentially decaying correlations, this real-space/Fourier-space pair is (note: although this is expressed in the scalar quantities $r=|r_{i}-r_{j}|$ and k=|k|, full *d*-dimensional integrals must be performed to obtain the correct normalizations):

(A3) $$C_{exp}\left(r\right)=\exp\left(-\frac{r}{\lambda }\right)⟺P_{exp}\left(k\right)=\frac{\Gamma \left(\frac{d+1}{2}\right)}{\pi^{\frac{d+1}{2}}}\times \frac{{\left(2\pi \right)}^{d}\left(1/\lambda \right)}{{\left({\left(1/\lambda \right)}^{2}+k^{2}\right)}^{\frac{d+1}{2}}}$$

and for Gaussian decaying correlations, the pair is:

(A4) $$C_{Gauss}\left(r\right)=\exp\left(-\frac{r^{2}}{2\lambda^{2}}\right)⟺P_{Gauss}\left(k\right)=\sqrt{{\left(\lambda^{d}2\pi \right)}^{2}}\exp\left(-\frac{{\left(k\lambda \right)}^{2}}{2}\right)$$

The value of λ then controls the *characteristic length scale* of the correlations and must be provided.

The task is then to create a GRF with the desired power spectrum. This can be done by first generating an initial GRF, $\phi_{0}\left(r\right)$, that has a power spectrum of 1:

(A5) $${\left|\hat{\phi_{0}}\left(k\right)\right|}^{2}=1$$

and then multiplying in reciprocal space that initial field by the square root of the intended final power spectrum:

(A6) $${\hat{E}}_{b}\left(k\right)=\sqrt{P(k)}\hat{\phi_{0}}\left(k\right)$$

Thus, by simple construction, the final field will have the correct power spectrum:

(A7) $$|{\hat{E}}_{b}{\left(k\right)|}^{2}=P\left(k\right){\left|\hat{\phi_{0}}\left(k\right)\right|}^{2}=P\left(k\right)$$

The final step is to perform an inverse discrete Fourier transform (IDFT) of the new field back to real-space to yield the desired correlated field. However, there is one additional issue related to the range of *k*s considered. The power spectrum as defined by the WK theorem in the discrete case runs from $-k_{max}$ to $k_{max}$, where $k_{max}=2\pi \left(N/2\right)/N$. Conversely, an IDFT, as defined in almost all numerical libraries, assumes a *k* running from 0 to 2π(N−1)/N. Thus, in order to perform the correct inverse Fourier transform, the *k* values should be shifted by N/2. In most numerical environments, there are dedicated functions for doing this—for example, fftfreq in numpy—and it is a necessary step.

The only remaining task is the generation of the initial field $\phi_{0}$ itself. This can be done in one of two ways. The first way is to exploit the fact that an uncorrelated field in real-space has a constant power spectrum:

(A8) $$C_{uncorr}\left(r\right)=\delta \left(r\right)⟺P_{uncorr}\left(k\right)=1$$

where δ(r) is the Dirac-delta function.

Thus, one can generate a Gaussian random number for each mesh point in real-space and then perform a DFT to yield a reciprocal-space field $\phi_{0}$ with the desired unit power spectrum. Alternatively, one can directly generate the field in reciprocal space by producing a randomized field with unit (complex) amplitude:

(A9) $$\phi_{0}\left(k\right)=\exp\left(2\pi U(0,1)\right)$$

where U(0,1) is a random number generated from a uniform distribution between 0 and 1. Thus, this randomized reciprocal-space vector has a trivial squared amplitude of 1. This approach ultimately works in that, when transformed back to real space, a Gaussian distribution results due to the central limit theorem, and it saves one from performing one extra numerically expensive Fourier transform.

Ultimately, the final field $E_{b}\left(r\right)$ should be real, and one can be very careful in the Fourier transform definitions and indexing to ensure a real result. One must also be careful that all the correct normalizations are used in the forward and inverse transforms such that the act of taking only the real component has the correct final standard deviation. However, a simpler and ultimately more robust implementation is to take the real part of the final field and then simply rescale its standard deviation to the desired final value. In addition to this, the previously discussed procedure assumes a mean field of zero; thus, the final field must simply be shifted to produce a non-zero mean. Thus:

(A10) $${\left[E_{b}\left(r\right)\right]}_{final}=\frac{E_{b}\left(r\right)}{\mathrm{std}(E_{b}\left(r\right))}\times \sigma_{E_{b}}+\langle E_{b}\rangle$$

The procedure detailed here is then what is stated in a concise algorithmic form in Section 2.1.

## Appendix B. Proof That the Exponential Band-Tail Integral Can Be Represented as a Hypergeometric Function

In this appendix, we show that the expression for the charge density of a band-tail with an exponentially decaying energy profile

(A11) $$n_{bound}\left(r,E_{F},T\right)=\sum_{i}\int_{-\infty }^{E_{i}\left(r\right)}{\mathrm{DOS}}_{bound}\left(r,E\right)/\left(1+\exp\left(\frac{E-E_{F}}{k_{B}T}\right)\right)dE$$

where

(A12) $${\mathrm{DOS}}_{bound}\left(r,E\right)=\sum_{i}N_{m,i}\exp\left(\frac{E-E_{i}\left(r\right)}{k_{B}T_{0,i}}\right),\;E<E_{i}\left(r\right)$$

can be rewritten as

(A13) ∑iNm,i(kBT0,i)×F121,1α,1α+1,−expEi(r)−EFkBT0,iα,α=kBT0,ikBT

where

(A14) F12a,b,c,x=Γ(c)Γ(b)Γ(c−b)∫01tb−1(1−t)c−b−1(1−xt)−adt

is a Gaussian hypergeometric function, and Γ(x) is the Gamma function. This transformation is essential to the analytical approach used in Section 2.4.1.

To minimize extraneous notation, here we will only assume a single band-tail (i.e., remove the sum over *i*). This does not affect the generality, as for multiple band-tails, one simply evaluates the hypergeometric function for each one independently and sums them. Thus, the integral under consideration is:

(A15) $$n_{bound}\left(r,E_{F},T\right)=N_{m}\int_{-\infty }^{E_{i}\left(r\right)}\frac{\exp\left(\frac{E-E_{i}\left(r\right)}{k_{B}T_{0}}\right)}{1+\exp\left(\frac{E-E_{F}}{k_{B}T}\right)}dE$$

If we write both the numerator and denominator as deviations from $E_{F}$ (i.e., in terms of $\left(E-E_{F}\right)$), we get:

(A16) $$N_{m}\int_{-\infty }^{E_{i}\left(r\right)}\frac{\exp\left(\frac{E-E_{F}+E_{F}-E_{i}\left(r\right)}{k_{B}T_{0}}\right)}{1+\exp\left(\frac{E-E_{F}}{k_{B}T}\right)}dE=N_{m}\exp\left(\frac{E_{F}-E_{i}\left(r\right)}{k_{B}T_{0}}\right)\int_{-\infty }^{E_{i}\left(r\right)}\frac{\exp\left(\frac{E-E_{F}}{k_{B}T_{0}}\right)}{1+\exp\left(\frac{E-E_{F}}{k_{B}T}\right)}dE$$

If we now define the function in the numerator as $z=\exp\left(\left(E-E_{F}\right)/k_{B}T_{0}\right)$, then the function in the denominator becomes

(A17) $$\exp\left(\frac{E-E_{F}}{k_{B}T}\right)=\exp\left(\left(\frac{E-E_{F}}{k_{B}T_{0}}\right)\times \left(\frac{k_{B}T_{0}}{k_{B}T}\right)\right)={\left[\exp\left(\frac{E-E_{F}}{k_{B}T_{0}}\right)\right]}^{\alpha }=z^{\alpha }$$

where $\alpha \equiv k_{B}T_{0}/k_{B}T$. The change of variables also requires a change in the infinitesimal of integration and the limits:

(A18)z(E=Ei(r))=exp(Ei(r)−EF)/kBT0(A19)z(E=−∞)=exp(−∞)−EF)/kBT0=exp−∞=0(A20)dzdE=exp(E−EF)/kBT0kBT0=zkBT0(A21)→dE=kBT0zdz

Making all of these substitutions yields the final result:

(A22) $$n_{bound}\left(r,E_{F},T\right)=\frac{N_{m}k_{B}T_{0}}{z_{0}}\int_{0}^{z_{0}}\frac{1}{1+z^{\alpha }}dz,\;z_{0}=\exp\left(\frac{E_{i}\left(r\right)-E_{F}}{k_{B}T_{0}}\right)$$

The task then switches to casting this integral as a Gaussian hypergeometric function.

To now write things in a form like Equation (A14), we should construct a new integration variable that both absorbs $z^{\alpha }$ into a single variable and is scaled such that the upper integration bound, $z_{0}$, is 1 in the new variable. The following meets these conditions:

(A23) $$t=\frac{z^{\alpha }}{z_{0}^{\alpha }}$$

The change of variable for this new variable then progresses like:

(A24)z=z0αt1α=z0t1α(A25)t(z=z0)=1(A26)t(z=0)=0(A27)dtdz=αzα−1z0α=αz0t1αα−1z0α=αz0α−1tα−1αz0α=αtα−1αz0(A28)→dz=z0αtα−1αdt

Thus, the integral becomes:

(A29) $$\int_{0}^{z_{0}}\frac{1}{1+z^{\alpha }}dz=\int_{0}^{1}\frac{1}{1+z_{0}^{\alpha }t}\left(\frac{z_{0}}{\alpha t^{\frac{\alpha -1}{\alpha }}}dt\right)=\frac{z_{0}}{\alpha }\int_{0}^{1}{\left(1-\left(-z_{0}^{\alpha }\right)\right)}^{-1}t^{\frac{1}{\alpha }-1}dt$$

By inspection and comparison with the definition of the hypergeometric function, we can then make the identification:

(A30)x=−z0α(A31)a=1(A32)b−1=1α−1→b=1α(A33)c−b−1=0→c=1α+1

And thus, we have that

(A34) z0αΓ1αΓ(1)Γ1α+1F121,1α,1α+1,−expEi(r)−EFkBT0α=z0α∫011−−z0α−1t1α−1dt

which, using the fact that Γ(1)=1 and Γ(x+1)=xΓ(x), the coefficient on the left-hand side becomes

(A35) $$\left(\frac{z_{0}}{\alpha }\right)\left(\frac{\Gamma \left(\frac{1}{\alpha }\right)\Gamma \left(1\right)}{\Gamma \left(\frac{1}{\alpha }+1\right)}\right)=\left(\frac{z_{0}}{\alpha }\right)\left(\frac{\Gamma \left(\frac{1}{\alpha }\right)}{\left(\frac{1}{\alpha }\right)\Gamma \left(\frac{1}{\alpha }\right)}\right)=\left(\frac{z_{0}}{\alpha }\right)\left(\alpha \right)=z_{0}$$

Plugging this in to Equation (A22) then gives the final expression:

(A36) nbound(r,EF,T)=NmkBT0×F121,1α,1α+1,−expEi(r)−EFkBT0α,α=kBT0kBT

Thus, it has been demonstrated.
